# The potential buffering role of self-efficacy and pain acceptance against invalidation in rheumatic diseases

**DOI:** 10.1007/s00296-017-3859-2

**Published:** 2017-10-30

**Authors:** Nigel Cameron, Marianne Kool, Fernando Estévez-López, Isabel López-Chicheri, Rinie Geenen

**Affiliations:** 1 Department of Psychology, Utrecht University, Heidelberglaan 1, 3584CS Utrecht, The Netherlands; 2 Association of Dutch Burn Centres, Beverwijk, The Netherlands; 3 Department of Physical Education and Sport, Faculty of Sport Sciences, University of Granada, Granada, Spain; 4 Faculty of Health Sciences, UCAM Catholic University of Murcia, Murcia, Spain

**Keywords:** Acceptance, Acceptance and commitment therapy, Invalidation, Psychological adjustment, Rheumatic diseases, Self-efficacy

## Abstract

A substantial amount of people with a rheumatic disease perceive invalidation consisting of lack of understanding and discounting (negative social responses). To get insight into the potential buffering role of self-efficacy and pain acceptance against invalidation, this cross-sectional study examined associations between these variables. Spanish speaking people (*N* = 1153, 91% female, mean age 45 ± 11 years) with one or multiple rheumatic diseases completed online the Illness Invalidation Inventory, the Chronic Pain Acceptance Questionnaire, and the Chronic Disease Self-Efficacy Scale. Higher self-efficacy (*t* = − 4.80, *p* = < 0.001) and pain acceptance (*t* = − 7.99, *p* = < 0.001) were additively associated with discounting. Higher self-efficacy (*t* = − 5.41, *p* = < 0.001) and pain acceptance (*t* = − 5.71, *p* = < 0.001) were also additively associated with lack of understanding. The combined occurrence of high self-efficacy and high acceptance was associated most clearly with lower lack of understanding (interaction: *t* = − 2.12, *p* = 0.034). The findings suggest the usefulness of examining whether interventions aimed at increasing self-efficacy and pain acceptance can help people with rheumatic diseases for whom invalidation is a considerable burden.

## Introduction

Invalidation is a common experience in people with a rheumatic disease. It includes non-acceptance by others, misunderstanding, disbelief, rejection, stigmatization, and suspicion that the problem is exaggerated or psychological [1]. Two major dimensions of invalidation have been identified: lack of understanding and discounting [2]. Lack of understanding reflects a lack of positive responses such as not recognizing, not comprehending, and not emotionally supporting the person. Discounting represents negative social responses and social rejection, including disbelieving, admonishing, dismissing inability to work, not acknowledging symptom fluctuations, and offering unusable advice. In rheumatic diseases, invalidation is related to worse physical and mental health [3, 4]. Pain and fatigue, which are the main symptoms in rheumatic diseases, are mostly not observable [5]. Therefore, symptoms and the consequent burden in people with rheumatic diseases are often poorly acknowledged and understood by others.

Invalidation depends on both the response of others to a person (objective invalidation) and on the appraisal that the person makes of such a response (subjective invalidation). To reduce objective invalidation, people near the patient should not deny the existence of what cannot be observed and they should not lecture, patronize, or overprotect the person. Instead they should listen, try to understand, and acknowledge the disorder and the person, and help (instrumental), comprehend and emotionally support the person. However, severity of invalidation not only depends on the actual invalidation by the social environment but also on the perception and skills of the person. This article focuses on subjective evaluation of invalidation and on skills that may help people to experience less invalidation.

To support people in dealing with adversities in life, management options are commonly derived from two broad treatment modalities: classical cognitive behavioral therapy (CBT) and acceptance and commitment therapy (ACT). CBT aims at increasing coping skills by promoting helpful thoughts and behavior [6]. ACT aims at accepting difficulties and to be committed to make changes in daily life that are in agreement with one’s life values [7]. Among the individual differences that may influence the experience of invalidation, self-efficacy [8] and acceptance [9, 10] skills are more or less prototypical for these CBT and ACT modalities, respectively. In the current study, self-efficacy and acceptance are operationalized as chronic disease self-efficacy and pain acceptance.

Self-efficacy is the personal belief that one can successfully perform particular behaviors to achieve a goal [11], such as managing pain [8] or managing daily challenges [12] and the consequences of a rheumatic disease [13–15]. Chronic disease self-efficacy has been related to less health distress, illness intrusiveness, activity limitation, depression and fatigue [as summarized in 16]. In analogy to these observed correlations, it is our hypothesis that self-efficacy to deal with a chronic disease is also related to less invalidation.

Pain acceptance, entails an active willingness to engage in meaningful activities in life despite having pain, and a reduction of unsuccessful attempts to avoid or control pain [17]. Greater pain acceptance has been related to less attention to pain [18], lower levels of pain-related anxiety and avoidance, less depression, and less physical and psychosocial disability [19–21]. Acceptance was also indicated to buffer the effect of negative affect on pain levels [9]. We chose pain acceptance as a measure of acceptance, because for any person with fibromyalgia, chronic pain is a primary symptom one needs to deal with, and a questionnaire to measure pain acceptance is available [22]. People who are able to accept their pain are assumed to be less in need of validation by others. Therefore, we hypothesize that people who accept their pain more are less likely to experience invalidating responses by others.

People who have a flexible repertoire of abilities are assumed to best deal with different kinds of unsatisfactory situations [23]. High coping skills such as reflected in chronic disease self-efficacy will make people feel competent to deal with situations in which control of invalidation is possible, while high acceptance skills will help people to actively and in full awareness experience even adverse situations without unnecessary attempts to change their frequency or form [7], which is a desirable skill when situations are hard to control.

Therefore, the aim of the present study was to examine the association of self-efficacy and pain acceptance with invalidation in people with a rheumatic disease. We hypothesized that high chronic disease self-efficacy and high pain acceptance are additively associated with low invalidation. Moreover, we hypothesized that people with both high chronic disease self-efficacy and high pain acceptance will experience a particularly low level of invalidation, which will be shown in a significant prediction of invalidation from the interaction between chronic disease self-efficacy and pain acceptance. Our cross-sectional design only allows correlational analyses and interpretations. We do not have the intention of establishing or implying causality. However, if the present study shows that self-efficacy and pain acceptance are associated with less invalidation, this will stimulate the design of prospective studies and therapies to test whether these factors help in dealing with invalidation.

## Methods

### Participants and procedure

The study was conducted according to the principles of the Declaration of Helsinki [24] and approved by the medical ethical review board of the University Medical Center Utrecht (Utrecht, The Netherlands). The study is part of an international online research project on invalidation in rheumatic diseases [25]. Participants were invited to take part in an online survey via a recruitment notice on websites of patient associations for rheumatic diseases located in various nations. The recruitment notice included information about the aim and content of the study, inclusion criteria, duration of participation (about 20 min), confidentiality, and a hyperlink to the online questionnaire. Participants could decide to participate after being informed about the study and were able to stop at any point if they desired to do so.

Each language version of the online research project had different questionnaires included next to the standard questionnaire package. The Spanish version included the questionnaire ‘Chronic Pain Acceptance Questionnaire (CPAQ)’ and ‘Spanish Chronic Disease Self-Efficacy Scale (SEMCD-S)’ to be able to study these concepts in relation to invalidation.

Inclusion criteria were (1) to report being diagnosed of at least one rheumatic disease by a health professional, (2) to be at least 18 years old, and (3) to have invalidation (discounting and lack of understanding) scores from at least three out of five sources of invalidation. This was done because calculating a mean invalidation score for one or two sources would have been less reliable [4]. Of the initial 1623 people of Spanish speaking countries who started with the online questionnaire, 1153 participants (71%) were included. The 470 participants were excluded for the following reasons: for 199 participants it was unknown whether the rheumatic disease was diagnosed by a health professional, 20 participants were younger than 18 years, 85 participants did not fill out the invalidation questionnaire, 136 participants had filled out less than three sources of the invalidation questionnaire, and 30 participants had submitted the questionnaires two or more times as was shown by a similar e-mail address, IP address, and year of birth.

Demographic and health-related characteristics of the sample are shown in Table 1. Most participants were female (91%) and with an average age of 45.4 (range = 18–82) years. Our dataset included the total years of received education since the age of 4. Education levels were estimated using the number of years it commonly takes to pass each level of the Spanish education system: primary level 1–8 years, secondary level 9–14 years, and tertiary level > 14 years.

**Table 1:** Characteristics of participants (*N* = 1153)

Gender: female, *n* (%)	1045 (90.6)
Gender: male, *n* (%)	108 (9.4)
Age, mean (SD) years	45.4 (10.5)
Education level, *n* (%)	
Primary, 1–8 years education after 4 years of age	7 (0.6)
Secondary, 9–14 years education after 4 years of age	377 (32.7)
Tertiary, > 14 years education after 4 years of age	769 (66.7)
Invalidation (3*I), mean (SD)	
Discounting	2.64 (0.82)
Lack of understanding	2.60 (0.81)
Self-efficacy (SEMCD-S), mean (SD)	4.78 (2.23)
Pain acceptance (CPAQ), mean (SD)	2.84 (0.83)
Rheumatic disease, *n* (%)	
Fibromyalgia	388 (33.7)
Rheumatoid arthritis	111 (9.6)
Ankylosing spondylitis	63 (5.5)
Systemic lupus erythematosus	124 (10.8)
Sjögren’s syndrome	35 (3.0)
A single other rheumatic disease	69 (6.0)
Fibromyalgia and another rheumatic disease	258 (22.4)
Multiple rheumatic diseases; not fibromyalgia	105 (9.1)

*SD* standard deviation, *SEMCD-S* Spanish Chronic Disease Self-efficacy Scale, *CPAQ* Chronic Pain Acceptance Questionnaire, 3*I Illness Invalidation Inventory

### Instruments

The Illness Invalidation Inventory [3*I; 2] was used to measure the degree of invalidation on the two domains ‘Discounting’ and ‘Lack of Understanding’ using the same eight items to refer to five potential sources of invalidation: Spouse, Family, Medical professionals, Work, and Social services. An example item is ‘My family thinks I should be tougher’. The items of the 3*I are answered on a 5-point Likert scale ranging from ‘Never’ (1) to ‘Very often’ (5). A high score means a high level of invalidation. The internal consistency of the 3*I can be classified as good [2, 26]. In the current study, the internal consistency was high for the sources of lack of understanding (three items, Spouse; *α* = 0.85, Family; *α* = 0.85, Medical professionals; *α* = 0.86, Work; α = 0.78, Social services; α = 0.86) and discounting (five items, Spouse; *α* = 0.75, Family; *α* = 0.81, Medical professionals; *α* = 0.82, Work; *α* = 0.85, Social services; *α* = 0.88). If a particular source of invalidation did not apply to them during the past year (e.g., because they did not have a spouse or work), then the patient was instructed to skip that part of the questionnaire. Mean scores for lack of understanding and discounting were calculated across the (at least three) available sources of invalidation [4].

The SEMCD-S [16, 27] measures self-efficacy in people with a chronic disease. The Spanish version consists of four items instead of the original six items, because the internal consistency was higher when excluding two items with the lowest item-to-scale correlations [28]. An example of an item is: ‘How confident are you that you can keep the fatigue caused by your disease from interfering with the things you want to do?’ Items are rated on an 11-point Likert scale ranging from 0 (never true) to 10 (always true). A high score reflects a higher level of self-efficacy. Reliability and validity of SEMCD-S were indicated to be good [16]. Internal consistency in the current study was very good (*α* = 0.91).

The brief 20-item version of the CPAQ [22] was used to measure pain acceptance. The questionnaire includes two dimensions: The Activity Engagement subscale (11 items) measures participation in daily activities while acknowledging the presence of pain, and the Pain Willingness subscale (9 items) that measures the degree to which pain is allowed in experience without efforts to avoid or control it. In the current study, to prevent that the model is obstructed by shared variance of two correlated predictor variables and to be able to compare one score that is characteristic for pain acceptance with one self-efficacy score, we used the total score for pain acceptance, i.e., the sum of the scores at both dimensions. Example items are: ‘My life is going well, even though I have chronic pain’ and ‘I need to concentrate on getting rid of my pain’ (reversed sign). The items are rated on a 7-point Likert scale from 0 (never true) to 6 (always true). A high score means a high acceptance of pain. Reliability and validity of the CPAQ subscales have been indicated to be good [22]. Internal consistency of the total score for pain acceptance in the current study was good (*α* = 0.79).

### Cutoff values for invalidation, self-efficacy, and pain acceptance

To describe participants’ levels of invalidation, self-efficacy, and pain acceptance as either ‘high’ or ‘low’, cutoff values were set by the authors at the middle of the response scales: 3.0 for discounting and lack of understanding (invalidation), 5.5 for self-efficacy, and 3.0 for pain acceptance. Lower scores on the two dimensions of invalidation and higher scores on self-efficacy and pain acceptance are more favorable.

### Statistical analyses

Statistical analyses were done with the Statistical Package for Social Sciences (SPSS version 21.0). Significance levels were set at *p* < 0.05 (two-tailed). The residual plots in regression analyses showed that the assumptions of linearity and normality were met (data not shown).

To test our hypotheses that self-efficacy, pain acceptance, and the interaction of self-efficacy and pain acceptance are associated with low invalidation, multiple hierarchical regression analyses were performed for discounting and lack of understanding separately. In Block 1, the demographic variables age, education level and gender were entered. Centered scores of self-efficacy and pain acceptance were entered in Block 2. In Block 3, the self-efficacy × pain acceptance interaction was entered, and in Block 4, fibromyalgia was entered to check whether results were not due to having a fibromyalgia diagnosis, because invalidation is experienced more often by people with fibromyalgia than by people with other rheumatic diseases [2].

## Results

### Percentage deviating scores

**Fig. 1:**
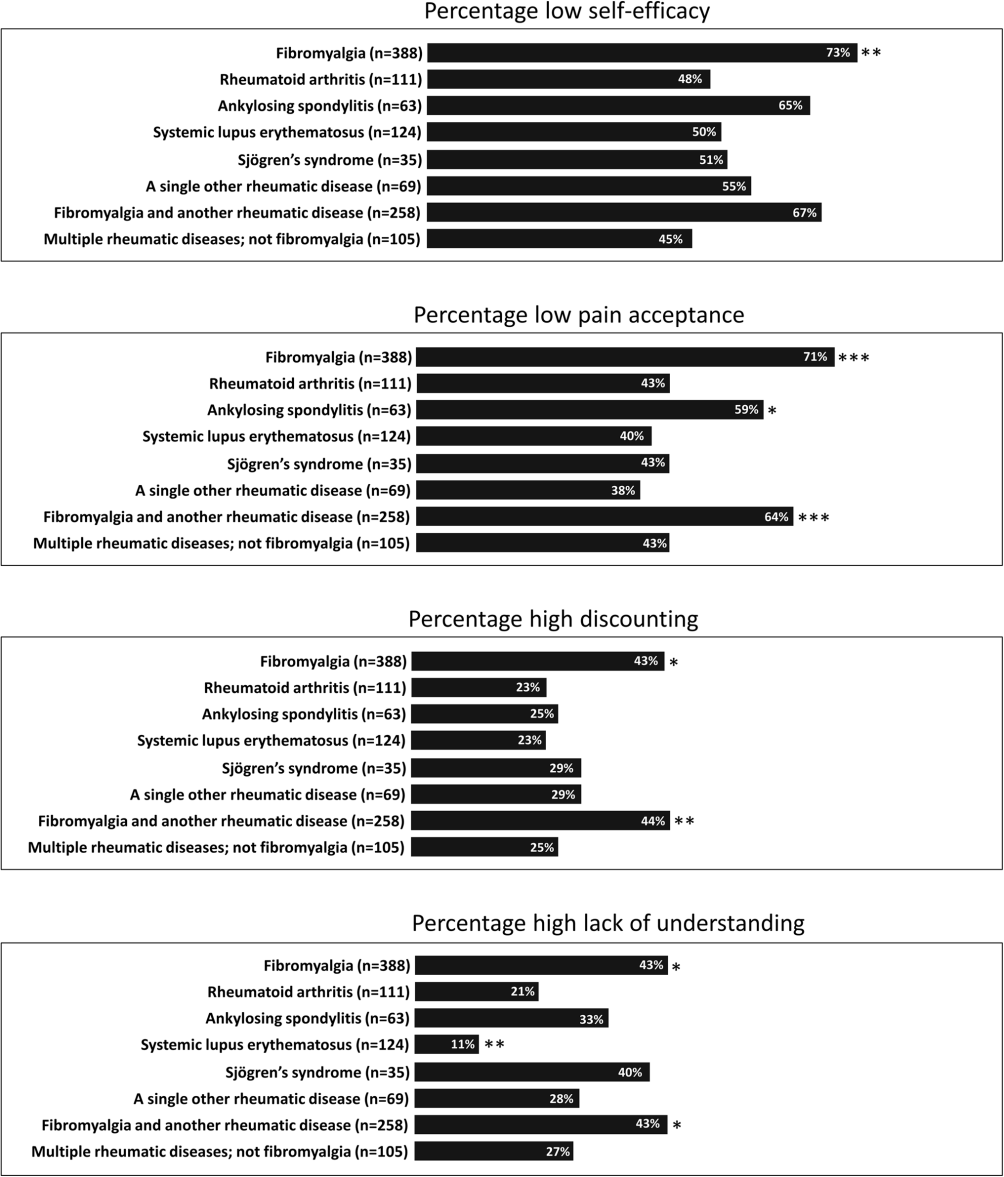
Percentages of participants with low self-efficacy, low pain acceptance, high discounting, and high lack of understanding per rheumatic disease; *p < 0.05, ** *p* < 0.01, *** *p* < 0.001

Figure 1 depicts percentages of people with low self-efficacy, low pain acceptance, high discounting, and high lack of understanding per specific rheumatic disease.

### Hierarchical regression analyses

Table 2 shows the results of the hierarchical regression analyses examining whether both dimensions of invalidation separately (discounting and lack of understanding) were associated with gender, age, education, self-efficacy, pain acceptance, the interaction of self-efficacy and pain acceptance, and fibromyalgia. The regression model for discounting showed that, in Block 1, gender and education were not associated with discounting while a higher age was associated with lower discounting (*t* = − 2.03, *p* = 0.042). In Block 2, higher levels of self-efficacy (*t* = − 4.80, *p* = < 0.001) and pain acceptance (*t* = − 7.99, *p* = < 0.001) were both additively associated with lower levels of discounting. One unit increase on self-efficacy and pain acceptance were associated with 0.06 and 0.25 units decrease on discounting. In Block 3, the interaction of self-efficacy and pain acceptance had no significant association with discounting (*t* = − 1.79, *p* = 0.072). In Block 4, although fibromyalgia was associated with more discounting (*t* = 6.39, *p* = < 0.001), the relation of discounting with self-efficacy and pain acceptance remained intact after inclusion of the fibromyalgia diagnosis in the model.

**Table 2:** Hierarchical regression analyses predicting the two dimensions of invalidation, discounting and lack of understanding, from demographic variables, self-efficacy, pain acceptance, and fibromyalgia in 1153 people with rheumatic diseases

Variable	Discounting (3*I)			Lack of understanding (3*I)		
	*b*	*β*	Adj. *R* ^2^	*b*	*β*	Adj. *R* ^2^
Block 1			0.001			0.003
Gender	0.050	0.083		− 0.138	− 0.049	
Age	− 0.005*	0.002		0.004	0.055	
Education (low)	0.181	0.312		0.178	0.017	
Education (high)	0.036	0.052		0.061	0.035	
Block 2			0.131***			0.102***
Gender	0.020	0.077		− 0.156*	− 0.056	
Age	− 0.004	0.002		0.005*	0.067	
Education (low)	0.102	0.291		0.097	0.009	
Education (high)	0.100*	0.048		0.118*	0.069	
Self-efficacy (SEMCD-S)	− 0.057***	0.012		− 0.065***	− 0.178	
Pain acceptance (CPAQ)	− 0.254***	0.032		− 0.183***	− 0.187	
Block 3			0.132***			0.104*
Gender	0.018	0.077		− 0.159*	− 0.057	
Age	− 0.004	0.002		0.005*	0.067	
Education (low)	0.108	0.291		0.104	0.010	
Education (high)	0.099*	0.048		0.117*	0.068	
Self-efficacy (SEMCD-S)	− 0.056***	0.012		− 0.064***	− 0.174	
Pain acceptance (CPAQ)	− 0.253***	0.032		− 0.182***	− 0.186	
Self-efficacy × pain acceptance	− 0.020	0.011		− 0.024*	− 0.060	
Block 4			0.162***			0.136***
Gender	0.092	0.077		− 0.082	− 0.029	
Age	− 0.008***	0.002		0.002	0.021	
Education (low)	0.094	0.286		0.089	0.009	
Education (high)	0.101*	0.048		0.118*	0.069	
Self-efficacy (SEMCD-S)	− 0.047***	0.012		− 0.054***	− 0.149	
Pain acceptance (CPAQ)	− 0.216***	0.032		− 0.144***	− 0.147	
Self-efficacy × pain acceptance	− 0.021	0.011		− 0.025*	− 0.063	
Fibromyalgia	0.309***	0.048		0.320***	0.196	

Education level, number of years education after 4 years of age: primary 1–8 years (low), secondary 9–14 years (middle), tertiary > 14 years (high)

*SEMCD-S* Spanish Chronic Disease Self-efficacy Scale, *CPAQ* Chronic Pain Acceptance Questionnaire, 3*I Illness Invalidation Inventory

*p < 0.05, **p < 0.01, ***p < 0.001

**Fig. 2:**
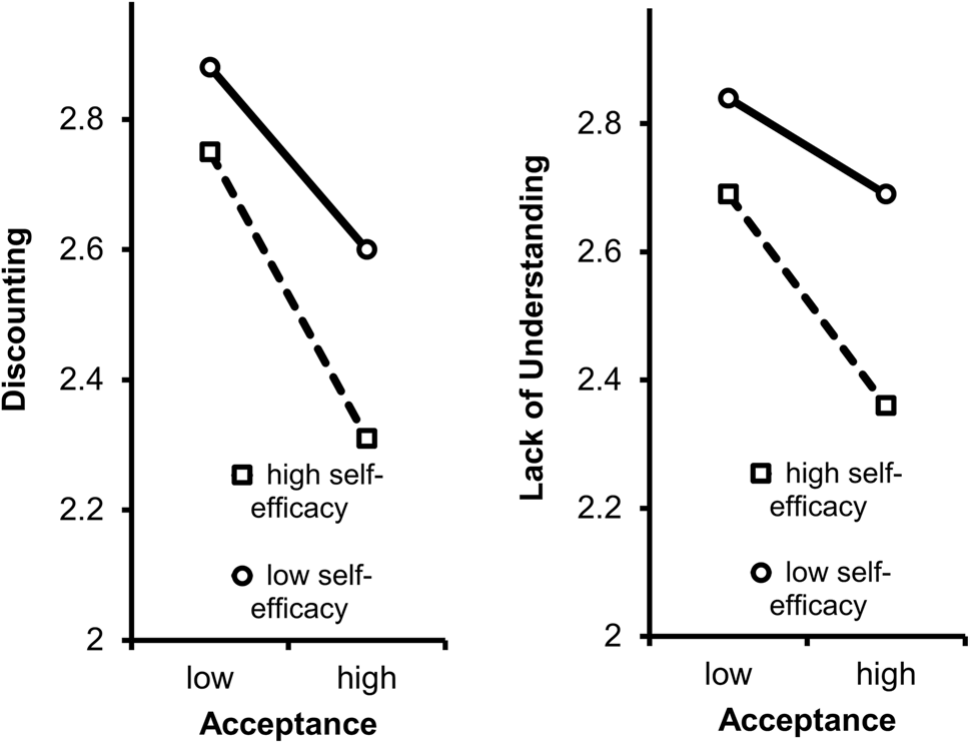
Invalidation (discounting and lack of understanding) predicted by self-efficacy and pain acceptance in 1153 people with rheumatic diseases

The regression model for lack of understanding showed that, in Block 1, neither gender nor age or education were associated with lack of understanding. In Block 2, higher levels of self-efficacy (*t* = − 5.41, *p* = < 0.001) and pain acceptance (*t* = − 5.71, *p* = < 0.001) were additively associated with lack of understanding. One unit increase on self-efficacy and pain acceptance were associated with 0.07 and 0.18 units decrease on lack of understanding. In Block 3, the interaction of self-efficacy and pain acceptance was associated with lack of understanding (*t* = − 2.12, *p* = 0.034). Figure 2 displays the interaction effect. The graph shows that particularly people who had both high self-efficacy and high pain acceptance perceived the least lack of understanding. In Block 4, although fibromyalgia was associated with higher lack of understanding (*t* = 6.55, *p* = < 0.001), the relation of discounting with self-efficacy and pain acceptance remained intact after the inclusion of fibromyalgia diagnosis in the model.

## Discussion

To establish whether both self-efficacy and pain acceptance may buffer against invalidation, our study examined the relationship of the two factors with invalidation. Our findings confirm that the level of invalidation is lower among people with rheumatic diseases with a higher level of either self-efficacy or pain acceptance. Moreover, people with both high self-efficacy and high pain acceptance showed even lower invalidation: the combination of high self-efficacy and high pain acceptance was significantly associated with low lack of understanding. This interaction was not significant for discounting.

Since the seminal work of Bandura [11] on self-efficacy, it has been shown that self-efficacy helps in dealing with numerous adversities [8, 12, 29] and that self-efficacy is an important component of self-management in chronic diseases [30] including rheumatic diseases [31]. Our correlational observation does not allow causal interpretations. The association may reflect that confidence to effectively deal with the disease and its consequences includes competency to deal with invalidation but it may also reflect that receiving less invalidation is positive for one’s confidence to deal with disease. In previous research, self-efficacy has been shown to be associated with self-management and the outcome of self-management programs [32, 33]. Moreover, a self-management program was shown to increase self-efficacy to manage pain [34]. This suggests that it is worth trying to reduce invalidation by increasing self-efficacy skills to deal with the disease and invalidation, which should be evaluated in future research.

**Fig. 3:**
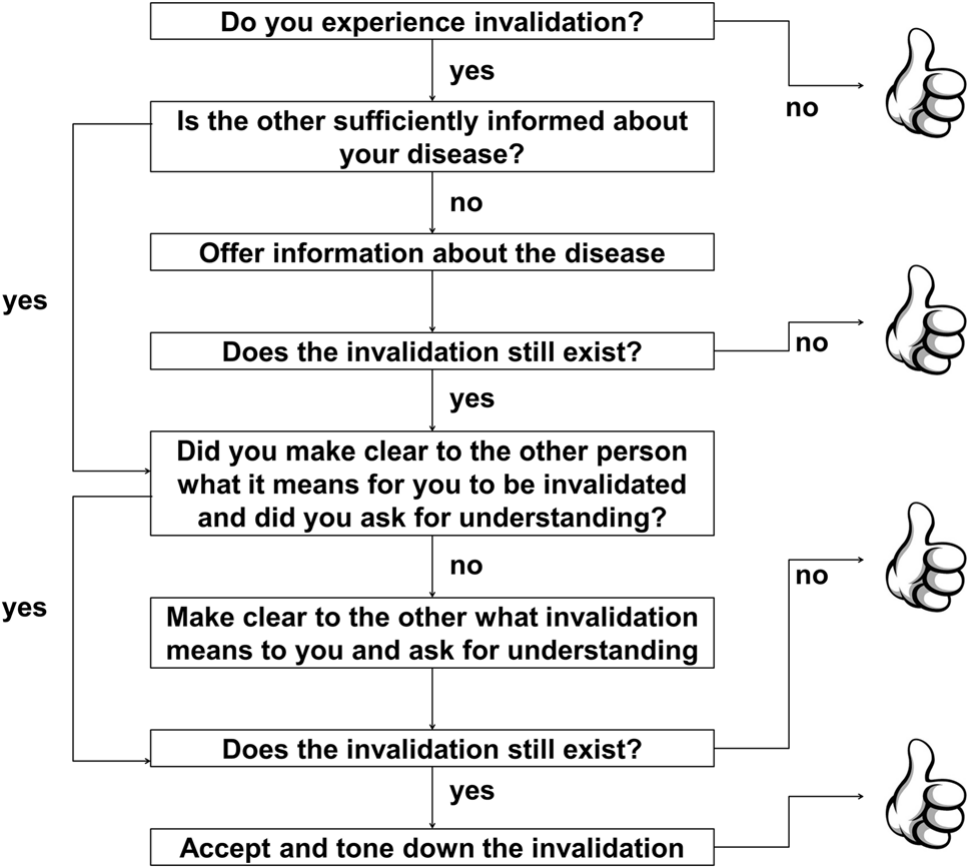
Recommendations to deal with invalidation (by Toon van Helmond, Sint Maartenskliniek, Nijmegen, The Netherlands)

Having taken account of chronic disease self-efficacy, our study showed that pain acceptance was additively associated with invalidation. Likely the higher scores on acceptance of pain reflect a general flexible willingness of people to have undesirable experiences without attempting to control them [35]. While the restructuring of cognitions and behavior is a fruitful approach to help people to deal with situations that can be changed, it is an asset if the acceptance of the inevitable consequences of the disease is part of people’s coping repertoire to deal with situations that cannot be changed [20, 36]. On the other hand, it is also possible that people who are able to accept their pain and pursuit their goals actually receive less invalidation from others. In recent years, Acceptance and Commitment Therapy [7] has been applied in the treatment of problems that may accompany chronic somatic diseases, including several rheumatic diseases [37]. This therapy helps people to accept the difficulties that come with a chronic disease and to be committed to make changes in daily life that are in agreement with one’s life values. Toon van Helmond (Sint Maartenskliniek, Nijmegen, the Netherlands) proposed a model of dealing with invalidation in which the first steps consist of communication and the final step involves acceptance as the best way to deal with invalidation when it is difficult to change the environment [38]: Fig. 3. Ideally, a person has the flexibility to deal effectively with invalidation as well as to accept it when the situation cannot be changed as was indicated by our finding that the combination of high chronic disease self-efficacy and high pain acceptance was associated with low lack of understanding.

In our study high invalidation, low chronic disease self-efficacy, and low pain acceptance were more prominent in fibromyalgia than in other rheumatic diseases (data analyses not shown), which was expected because of the invisibility of signs of the disease and its pathological substrate. However, in all disease groups, using the middle of the response scale as cutoff criterion, a minimum of 38% showed low pain acceptance, 45% low chronic disease self-efficacy, 23% high discounting, and 11% a high lack of understanding. This clearly shows that health care professionals should be alert to the burden of invalidation in all rheumatic diseases. Moreover, our data suggest that it is worth trying to decrease invalidation by stimulating chronic disease self-efficacy and pain acceptance skills in self-management training, educational materials, and interventions in patients with rheumatic diseases.

Although no causality can be inferred from the cross-sectional design of our study, the results are valuable because they give an indication of the prevalence of three aspects that are considered core to adjustment to chronic disease [5] and because the results indicate who might be better protected against invalidation. Future studies with a prospective or clinical experimental design are needed to clarify the causal direction of the observed associations. Furthermore, it would be worthwhile to examine more specifically whether patients with a rheumatic disease and high invalidation scores can be helped by acquiring skills of accepting invalidation (instead of or besides to pain acceptance) and by increasing self-efficacy regarding invalidation (instead of or besides self-efficacy in dealing with a chronic disease). A methodological limitation of the studied sample is that inclusion was based on self-reported diagnoses of rheumatic diseases without certification by a medical specialist. Moreover, the recruitment through the internet may have led to a lower representation of the older population and people with a low social economic class. Finally, people with fibromyalgia were overrepresented in this study. However, the findings in the regression analyses remained intact when the regressions were adjusted for a fibromyalgia diagnosis and demographic characteristics.

The current study shows that invalidation, low chronic disease self-efficacy, and low pain acceptance are prominent in fibromyalgia and other rheumatic diseases, and that invalidation is low in people with higher chronic disease self-efficacy and higher pain acceptance. Additionally, people with both higher chronic disease self-efficacy and pain acceptance experience the lowest lack of understanding. The findings suggest that it is useful to examine whether interventions aimed at increasing chronic disease self-efficacy and pain acceptance help people with rheumatic diseases for whom invalidation is a considerable burden.

## References

[1] Kool MB, van Middendorp H, Boeije HR, Geenen R (2009). Understanding the lack of understanding: invalidation from the perspective of the patient with fibromyalgia. Arthritis Rheum.

[2] Kool MB, van Middendorp H, Lumley MA, Schenk Y, Jacobs JWG, Bijlsma JWJ, Geenen R (2010). Lack of understanding in fibromyalgia and rheumatoid arthritis: the Illness Invalidation Inventory (3*I). Ann Rheum Dis.

[3] Ghavidel-Parsa B, Amir Maafi A, Aarabi Y, Haghdoost A, Khojamli M, Montazeri A, Sanaei O, Bidari A (2014). Correlation of invalidation with symptom severity and health status in fibromyalgia. Rheumatology.

[4] Kool MB, van Middendorp H, Lumley MA, Bijlsma JWJ, Geenen R (2013). Social support and invalidation by others contribute uniquely to the understanding of physical and mental health of patients with rheumatic diseases. J Health Psychol.

[5] Geenen R, Finset A, Bijlsma JWJ, da Silva JAP, Hachulla E, Doherty M, Cope A, Lioté F (2012). Psycho-social approaches in rheumatic diseases. EULAR textbook on rheumatic diseases.

[6] Keefe FJ, Abernethy AP, Campbell CL (2005). Psychological approaches to understanding and treating disease-related pain. Annu Rev Psychol.

[7] Hayes SC, Luoma JB, Bond FW, Masuda A, Lillis J (2006). Acceptance and commitment therapy: model, processes and outcomes. Behav Res Ther.

[8] Jackson T, Wang Y, Wang Y, Fan H (2014). Self-efficacy and chronic pain outcomes: a meta-analytic review. J Pain.

[9] Kratz AL, Davis MC, Zautra AJ (2007). Pain acceptance moderates the relation between pain and negative affect in female osteoarthritis and fibromyalgia patients. Ann Behav Med.

[10] Åkerblom S, Perrin S, Fischer MR, McCracken LM (2015). The mediating role of acceptance in multidisciplinary cognitive-behavioral therapy for chronic pain. J Pain.

[11] Bandura A (1977). Self-efficacy: toward a unifying theory of behavioral change. Psychol Rev.

[12] Schneider S, Junghaenel DU, Keefe FJ, Schwartz JE, Stone AA, Broderick JE (2012). Individual differences in the day-to-day variability of pain, fatigue, and well-being in patients with rheumatic disease: associations with psychological variables. Pain.

[13] Hermsen LAH, van der Wouden JC, Leone SS, Smalbrugge M, van der Horst HE, Dekker J (2016). The longitudinal association of cognitive appraisals and coping strategies with physical functioning in older adults with joint pain and comorbidity: a cohort study. BMC Geriatrics.

[14] Lee J, Lee K, Park D, Kim S, Nah S, Lee JH, Kim S, Lee Y, Hong S, Kim H, Lee H, Kim HA, Joung C, Kim S, Lee S (2017). Determinants of quality of life in patients with fibromyalgia: a structural equation modeling approach. PLoS One.

[15] Thombs BD, Kwakkenbos L, Riehm KE, Saadat N, Fedoruk C (2017). Comparison of self-efficacy for managing chronic disease between patients with systemic sclerosis and other chronic conditions: a systematic review. Rheumatol Int.

[16] Ritter PL, Lorig K (2014). The English and Spanish Self-Efficacy to Manage Chronic Disease scale measures were validated using multiple studies. J Clin Epidemiol.

[17] McCracken LM, Carson JW, Eccleston C, Keefe FJ (2004). Acceptance and change in the context of chronic pain. Pain.

[18] Viane I, Crombez G, Eccleston C, Devulder J, De Corte W (2004). Acceptance of the unpleasant reality of chronic pain: effects upon attention to pain and engagement with daily activities. Pain.

[19] Healy GM, Finn DP, O’Gorman DA, Maharaj C, Raftery M, Ruane N, Mitchell C, Sarma K, Bohacek M, McGuire BE (2015). Pretreatment anxiety and pain acceptance are associated with response to trigger point injection therapy for chronic myofascial pain. Pain Med.

[20] McCracken LM, Davies M, Scott W, Paroli M, Harris S, Sanderson K (2015). Can a psychologically based treatment help people to live with chronic pain when they are seeking a procedure to reduce it?. Pain Med.

[21] Trompetter H, Bohlmeijer E, Veehof M, Schreurs K (2015). Internet-based guided self-help intervention for chronic pain based on acceptance and commitment therapy: a randomized controlled trial. J Behav Med.

[22] Bendayan R, Esteve R, Blanca MJ (2012). New empirical evidence of the validity of the Chronic Pain Acceptance Questionnaire: the differential influence of activity engagement and pain willingness on adjustment to chronic pain. Br J Health Psychol.

[23] Vriezekolk JE, van Lankveld WGJM, Eijsbouts AMM, van Helmond T, Geenen R, van den Ende CHM (2012). The coping flexibility questionnaire (COFLEX). Development and initial validation in patients with chronic rheumatic diseases. Rheumatol Int.

[24] World Medical Association (WMA) Declaration of Helsinki. Seoul: WMA, October 2008. http://www.wma.net/en/20activities/10ethics/10helsinki/index.html. Accessed 8 Nov 2013

[25] Kool MB, van de Schoot R, López-Chicheri García I, Mewes R, Da Silva JAP, Vangronsveld K, Wismeijer AJA, Lumley MA, van Middendorp H, Bijlsma JWJ, Crombez G, Rief W, Geenen R (2014). Measurement invariance of the Illness Invalidation Inventory (3*I) across language, rheumatic disease and gender. Ann Rheum Dis.

[26] Kool MB, Geenen R (2012). Loneliness in patients with rheumatic diseases: the significance of invalidation and lack of social support. J Psychol.

[27] González VM, Stewart A, Ritter PL, Lorig K (1995). Translation and validation of arthritis outcome measures into Spanish. Arthritis Rheum.

[28] Lorig KR, Sobel DS, Ritter PL, Laurent D, Hobbs M (2001) Effect of a self-management program for patients with chronic disease. Eff Clin Pract 4:256–262. http://ecp.acponline.org/novdec01/lorig.pdf 11769298

[29] Schönfeld P, Brailovskaia J, Bieda A, Zhang XC, Margraf J (2016). The effects of daily stress on positive and negative mental health: mediation through self-efficacy. Int J Clin Health Psychol.

[30] Marks R, Allegrante JP, Lorig K (2005). A review and synthesis of research evidence for self-efficacy-enhancing interventions for reducing chronic disability: implications for health education practice (part II). Health Promot Pract.

[31] Ammerlaan JW, van Os-Medendorp H, de Boer-Nijhof N, Maat B, Scholtus L, Kruize AA, Bijlsma JWJ, Geenen R (2017). Preferences and needs of patients with a rheumatic disease regarding the structure and content of online self-management support. Patient Educ Couns.

[32] Miles CL, Pincus T, Carnes D, Homer KE, Taylor SJC, Bremner SA, Rahman A, Underwood M (2011). Can we identify how programmes aimed at promoting self-management in musculoskeletal pain work and who benefits? A systematic review of sub-group analysis within RCTs. Eur J Pain.

[33] Wilski M, Tasiemski T (2016). Illness perception, treatment beliefs, self-esteem, and self-efficacy as correlates of self-management in multiple sclerosis. Acta Neurol Scand.

[34] Damush TM, Kroenke K, Bair MJ, Wu J, Tu W, Krebs EE, Poleshuck E (2016). Pain self-management training increases self-efficacy, self-management behaviours and pain and depression outcomes. Eur J Pain.

[35] McCracken LM, Zhao-O’Brien J (2010). General psychological acceptance and chronic pain: there is more to accept than the pain itself. Eur J Pain.

[36] Vriezekolk JE, Eijsbouts AMM, Van Lankveld WGJM, Beenackers H, Geenen R, Van den Ende CHM (2013). An acceptance-oriented cognitive-behavioral therapy in multimodal rehabilitation: a pretest-posttest evaluation in highly distressed patients with rheumatic diseases. Patient Educ Couns.

[37] Hughes LS, Clark J, Colclough JA, Dale E, McMillan D (2017). Acceptance and commitment therapy (ACT) for chronic pain: a systematic review and meta-analyses. Clin J Pain.

[38] Kool MB, Geenen R (2016). Onbegrip en verbittering bij mensen met een aandoening (Invalidation and embitterment in people with a disease). PsyXpert.

